# Structural Determinants in Family Planning Service Utilization in Ethiopia: EDHS 2011 Analysis

**DOI:** 10.1155/2015/495745

**Published:** 2015-12-09

**Authors:** Jembere Gizachew Balew, Yongtae Cho, Clara Tammy Kim, Woorim Ko

**Affiliations:** Seoul National University, Republic of Korea

## Abstract

Family planning coverage has improved in Ethiopia in the last decade, though fertility is still about 5.8 in the rural setup. In this paper, the major structural determinants of family planning service were analyzed using a multilevel model from 8906 individual women observation in the 2011 EDHS data. The results show that there is a big variation in family planning use both at the individual and between group levels. More than 39% of the variation in FP use is explained by contextual cluster level differences. Most of the socioeconomic predictors; respondent's education, ethnicity, and partners' education as well as employment status and urbanization were found to be significant factors that affect FP use. Similarly health extension visit and media access were found to be strong factors that affect FP service at both individual and cluster levels. This evidence concludes that addressing these contextual factors is very crucial to strengthen FP use and fertility reduction in the nation, beyond individual behavioral changes.

## 1. Introduction

Since Malthus, and specifically since the 1994 Cairo conference, high fertility and fast population growth are perceived as one of the deterring factors for development [1–3]. Following which governments and international donors have put emphasis on fertility and FP issues in developing nations. However, during the last two decades, since 1995, the year following the Cairo conference, the percentage of total population assistance by donors to FP has declined from 55% to 9% [1, 4], probably due to the rise of HIV/AIDS or less attention to FP and SRH [1, 5], best exemplified by the US global gag rule [6].

Conceptually FP programs are believed to have a wider benefit than the traditional rhetoric of limiting or spacing childbirth [7, 8]. FP allows spacing of pregnancies in young women, who are at increased risk of health problems and death from early childbearing. This might contribute to reduction of fetal and infant problems which can further reduce infant mortality. By reducing rates of unintended pregnancies, FP can also reduce the need for unsafe abortion [9]. Poverty and hanger can be reduced through FP while at the same time averting more than 32% of all maternal deaths and nearly 10% of childhood deaths [10]. A study in Ethiopia has showed that FP would avert 12,782 maternal deaths and more than 1.1 million child deaths by 2015 [11].

Promisingly, in the past 40 years, family planning programs have played a major part in raising the prevalence of contraceptive from less than 10% to 60% and reducing fertility in developing countries from six to three births per woman [10]. Globally 63 percent of married women or those in union were using some form of contraception in 2011; however, still, an estimated 222 million women in developing countries would like to delay or stop childbearing but are not using any method of contraception [7]. But, in the WHO African Region, contraceptive prevalence was below 27 percent [1, 12] with more than 25% of married women or those in union in Sub-Saharan Africa having an unmet need for family planning [13].

Ethiopia with an estimated total population of over 90 million based on 2007 census projection [14, 15] has an annual population growth rate of 2.6 percent [16]. Its population is very young with a median age of 17 years and an estimated life expectancy at birth of 63 years [17]. By 2050, its population is projected to be more than 188 million [18]. According to the 2011 DHS survey, the total fertility is 4.8 and the contraceptive prevalence is 29 percent. Average household size is 4.6 with urban population having a lesser household size at 3.7 compared with 4.9 in the rural population. Since 1990s, FP service in Ethiopia is showing a promising achievement that intern helps to reduce fertility and lessen the burden of a fast growing dependent population. The first national population policy was put in place in 1993 [19]. This policy document with some other supporting documents like the national reproductive health strategy [20], FP manual, and the comprehensive safe abortion guideline in 2009 [21], health sector development plan [22] and the Criminal Code, Proclamation number 414/2004 [23], have played a key role in increasing access to SHR service in the country.

However, a study on proximate fertility determinants in Gondar [24] has revealed that postpartum infecundability, most from prolonged breastfeeding, is the strongest factor reducing fertility, while the index of nonmarriage and contraceptive is not well exploited. Nonetheless, a study by Teklu et al. showed that contraception is becoming a more important proximate determinant in rural areas; registering a reduction in its index from 0.97 to 0.83 from 2000 to 2011 [25].

In general, for a very long period, fertility has remained high and FP coverage has stagnated at its low coverage in most parts of Africa either due to a poorly fitting policy design or intervention that understands the structural determinants of fertility in these nations. Some of these factors include economic inequality and livelihood insecurity, poverty, gender inequality, lack of education, cultural and religious barriers, ethnic differences, urbanization, and others that might be context specific. Indeed there are overwhelming evidences confirming sociodemographic characteristics as being very important factors to affect FP utilization among Ethiopians. However, past studies in the country that involve health behavior had tried to approach factors that affect health utilization only at an individual level. However, human population has complex social arrangement where group behaviors can also affect individual's health behavior, which applies to FP service too. Hence, it is very important to see the effects of group behaviors and individual behaviors together to understand the complex nature of FP programs in the country for both individual and community based interventions and national level policy directions. This study has utilized a multistage data from EDHS 2011 to analyze group level context and individual factors that affect FP service using a mixed effects multilevel modeling.

## 2. Methodology

Before conducting a multilevel study design, understanding the community living arrangement and data structure is very important. The Ethiopian community is administratively structured under 11 regions and two city administrations basically based on ethnic classification. However, practically the way regions, ethnic, religious, and other social groupings are categorized does not have a clear demarcation. For instance, it is easy to notice that ethnic boundaries go beyond regional differences resulting in ethnic division that is not nested under region and vice versa.

**Figure 1:**
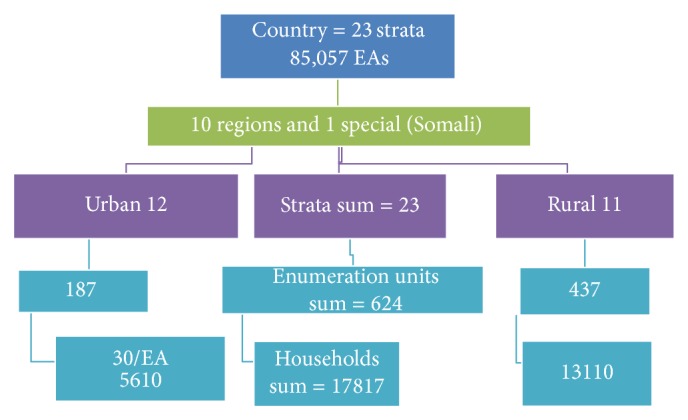
Two-stage cluster sampling and sampling data frame EDHS 2011.

The DHS data is already designed in a two-stage cluster data (Figure 1). Individual households are randomly selected out of randomly selected primary sampling units. These primary sampling units or clusters which include more than 30 households are parts of a Kebele or village (kebele is the smallest administrative unit and village is the smallest social unit under a given Kebele that does not have administrative role) where individual households are randomly selected from.

The primary sampling units are selected randomly [26]. We used sampling weights due to the nonproportionality of sampling methodology. Statistically these weights were included for further analysis using the “survey package” in R statistical program [27].

**Figure 2:**
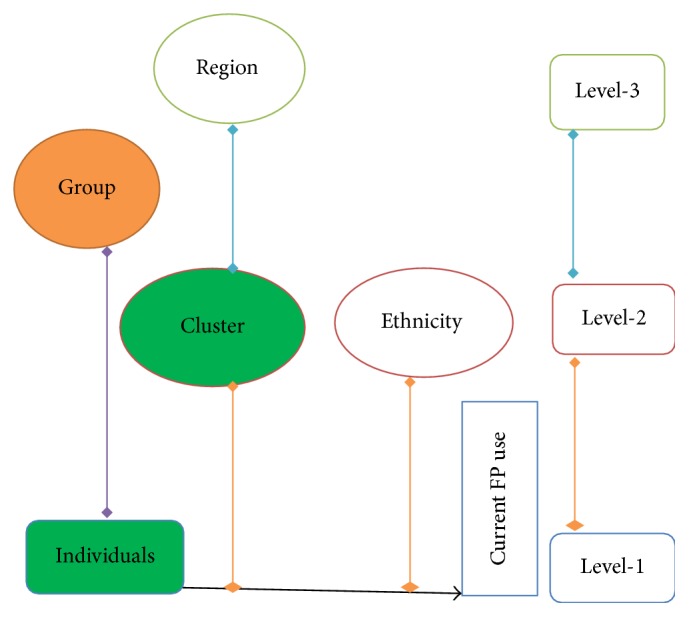
Multilevel structure of predictors of family planning utilization.

DHS questionnaires allow different units of analysis, households, household members, women, children, and couples being the major categories. This research is based on individual women's data which contains record for every eligible woman for the study. The model in this study has tried to explore possible group effects that might affect FP utilization (Figure 2). Finally for the purpose of this study a two-stage multilevel analysis that explores individual at level one and clusters at level two on FP practice was used to see the structural determinants of current FP utilization.

### 2.1. Data Source and Data

According to EDHS 2011 methodology, a nationally representative sample of about 18,500 households was selected and all women aged 15–49 and all men aged 15–59 in these households were eligible for the individual interview module of the survey; however, the final respondents for the Ethiopian 2011 survey were 16515. The SAS version of this data was then downloaded from the DHS website after getting approval through online registration. Out of these respondents, a reproductive age woman should be sexually active and nonpregnant and should not be postpartum abstaining to be eligible for FP service. This resulted in final 8906 observations for further analysis using R programming software [28].

### 2.2. Study Variables

The main dependent variable in this study is current FP utilization, a binary variable. This response is then modeled to historical predictor variables that were selected based on existing evidences (see Table 1). Except the variable cluster and some new variables aggregated from the existing predictors to indicate group level effects, most other predictors were assumed to affect FP consumption within a cluster. The aggregate values that are assumed to affect group level variation include income (proportion of richest income group), education (proportion of elementary level education), health extension visit (proportion of houses having health extension visit), and media (proportion of samples having better media access (level three and above out of six categories)). This helps us design both random intercept and random slope model at level two and explore the effects of the predictor variables at the individual and group level.

### 2.3. Basic Concepts of Multilevel Model

#### 2.3.1. Random Intercept Model

The random intercept model is used to model unobserved heterogeneity in the overall FP utilization by introducing random effects at a cluster level. In this model the intercept is the only random effect that the groups differ with respect to the average value of the response variable, but the relation between explanatory and FP utilization is assumed to be constant between clusters. Current FP utilization is a binary response variable coded as yes or no. Running a linear regression has multiple drawbacks for such a binary variable and transformation of the response variable needs a logistic link function. The resulting log odds value (logit(*p*_*ij*_/(1 − *p*_*ij*_))) is regressed on potential structural predictors as seen in the following model:

(1) log⁡pij1−pij=β0+β1x1+β2x2+⋯+βn1xn+uj+eij,

where *β*_0_ + *β*_1_ *x*_1_ + *β*_2_ *x*_2_ + ⋯+*βn*_1_ *x*_*n*_ + *e*_*ij*_ is the fixed part and *u*_*j*_ is the random part.

It is assumed that the residual *u*_*j*_ is mutually independent and normally distributed with mean zero and variance of *σ*_*u*_^2^.

#### 2.3.2. Random Slope Model

This is an extended method of multilevel analysis where individual predictor variables are assumed to vary across groups too. In a practical situation it is difficult to find predictors that do not vary across groups at a community level. Hence, it is important to fit some variables that are assumed to be varying across different clusters. The model looks like the following for a binary response variable:

(2) $$\begin{array}{c} \mathrm{logit}\left(\;\frac{p}{1-P}\;\right)=\beta_{0}+\beta_{1}x_{ij}+u_{1j}x_{ij}+\cdots +u_{0i}+e_{ij}. \end{array}$$

All the other components remaining constant, *u*_1*j*_ *x*_*ij*_, is the random slope term and *u*_0*i*_ is the cluster level error.

## 3. Objective of the Study

The major objective of this study is to analyze the structural determinants of FP service utilization in Ethiopia.

### 3.1. Hypothesis

1. Family planning utilization is positively affected by education and income status.
2. Women employment status improves better family planning service utilization.
3. Husband education and employment promote better FP service utilization.
4. Access to media services and community health services improve FP service utilization.
5. Structural factors have contextual effect on family planning service.

## 4. Results

This study uses a total of 8906 individual women's data from the 2011 Ethiopian demographic and health survey, excluding those who are not sexually active within the last month of interview for nonmenopausal reasons. The result shows that around 2495 (28%) women were current utilizers of FP.  Table 2 shows the disaggregation by social characteristics.

Age is one of the determining factors in most health services. A bivariate cross tabulation (Table 2) of FP utilization by age category shows a lower utilization in teenagers which improves after age of 20 and drops again after the age of 35. On the other hand, a woman with higher education has a sixfold odds of FP acceptance than those with no education while the variation with respect to regional difference ranges from 4.96 percent in Somali to 64.46 percent in Addis Ababa followed by Gambela (46.09%), Dire Dawa (38.66%), Harari (36.4%), and Amhara (34.73%).

**Figure 3:**
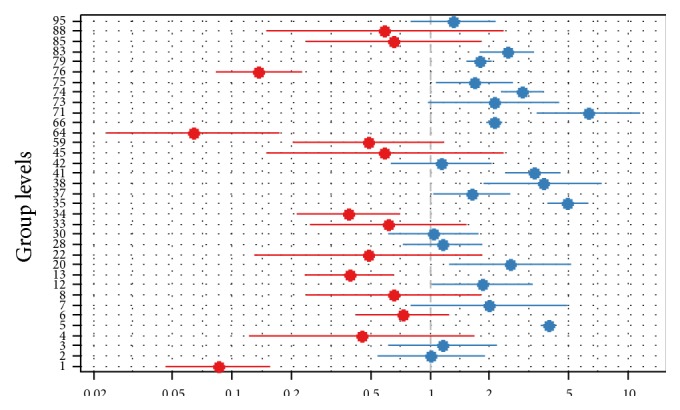
Random intercepts by ethnic grouping (the codes on the column represent ethnic codes for Ethiopia according to DHS 2011).

A further disaggregation of the data to ethnic level shows that people from Amhara origin have higher odds of FP use (OR = 2.00) than other ethnic groups. However, a detailed view of the specific ethnic variations shows that far lower from the average Neuer, Afar, Somali, Mejenger, and Derashe communities are found and higher from the average there are Keficho, Amhara, Guraghe, and Dauro communities (Figure 3) despite the higher variation due to the small sample size. The scale on the *x*-axis in this figure shows the random effect for each individual ethnic group in log odds scale.

Being urban and rural resident has also created a difference of about 54.4 percent in FP service utilization from 24.71 percent in rural resident to 54.18 percent in urban resident woman. From a religion perspective Orthodox Christians have about 36.72 percent FP practice, much better than other religions, 31.13% in other Christians, 20.57% in Muslims, and 11.19% in other religious followers.

There are ample studies that show the number of offspring as the other determining factor in fertility. In this study, women with one under one child are the highest consumers of FP [37.8%] while it shows a decline for mothers having two or more children. This proportion almost matches the effect of number of household members on FP consumption. FP use decreases as the number of household members' increases, which indeed might show the growing practice of FP utilization in newly married couples unlike the trend that has existed for long among their mothers, owing to improvements in educational status and other factors that change their behavior, but it needs to be statistically confirmed.

On the other hand, relation to household head that might point the level of access and authority the woman has to resources shows that being a sister or a daughter to the household head has the lowest rate of FP consumption. However, it is very important to understand how a married woman can be with her family members in the local setup. Traditionally in most Ethiopian cultures, a woman goes to her husband's family, where the husband usually resides. However, if separated or divorced or if the husband goes to her family (rarely), she will live with her father, or her brother or sisters and other relatives. The same reasoning can be applied to a woman living alone or being a head of a household in having a lower FP use.  Table 3 summarizes the descriptive result of a woman's family planning in relation to family size and husband characteristics.

However, all these differences might also be due to other socioeconomic differences. Those with low number of children are most newly married couples, having a better education and access to FP, while those having many children are already mature, having low level of education, with low FP need and access. This can be supported by the increase in literacy level at an early age as more youngsters are attending school unlike their mothers and other elder family members; these all need a further analysis to understand this complex relationship.

### 4.1. Results and Discussion from Random Intercept Model

A multilevel analysis of FP utilization using lme4 package [29] in R programing software was used to disentangle the statistical significance of each structural and behavioral determinant factor on FP. To visualize model fits the “jpPlot” package [30] was used (refer to (1)). As discussed in the methodology section, a logit transformation of the binary response of current FP service is modeled on the explanatory variables. The first null fit was a single level analysis using generalized linear model (GLM). This model is developed to evaluate the null hypothesis that there is no cluster level difference in FP service utilization after comparing the results of model fit with a null model for mixed effects design. Likely hood results which measure the probability of producing the observed values in FP utilization using the specified fits were used as measures of model fit.

Accordingly, as can be seen from the two model outputs in Table 4 the deviance from the single to the mixed logistic model has reduced with a chi square value of 1365.6 (df = 1), which indicates that the group level variation in FP is not zero. Or there is dependence among individuals within a cluster. This tells us that unlike the assumption of simple linear regression model, independence of individual observations is violated in our study. Meaning we have to use the multilevel model than the single level model to take into account the group level effects on FP service utilization taking into account the group level dependency.

**Figure 4:**
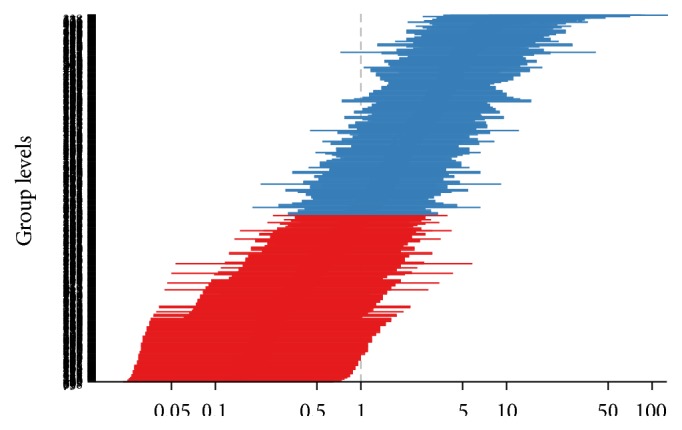
Caterpillar plot of cluster level random effects in log odds (the levels on the column are too many to visualize (650 clusters)).

Exploration of the random intercepts for FP utilization using caterpillar plot shows the presence of huge variation among each cluster (Figure 4). Note that the value in the *x*-axis is the variation of the cluster average (odds ratios of random intercept). The blue plot shows the clusters whose odds of FP are above the population one. The plot clearly shows the level of variation among individual clusters. The mean value which indicates the average FP consumption at a national level is estimated to be log odds of −1.23 which represents an average of 22.6% percent FP utilization, with a group level variance of 2.178 (a FP utilization of 3.2 percent and 84.8 percent from a null model), which is slightly lower than the CPR frequency (Table 2), where actually the variation is from 4.96 percent to 64.46 percent [31]. This shows that the model is not well fit, since it is a null model that needs further fitting.

The same graph points out that clusters which have odds of more than three in FP use are found in Addis Ababa, Amhara, Gambela, and Ben Gumuz, whereas the majority of Afar and Somali are found below 0.33, which is more than three standard deviations below the mean. Further review has revealed that certain clusters in Anyiwak community, Guraghe, Keficho, and some parts of Oromo community have better FP, which might be due to the small sample size or a real picture of better FP coverage that needs further quantitative and qualitative studies.

Exploration of variations within clusters using the variance partitioning for multilevel model shows that more than 39 percent of the variation in these observation is explained by the cluster level model. Note how the interclass correlation is calculated, keeping the individual level error constant, 3.29 [32]:

(3) VPC=Level2  residual variancelevel1  residual variance+Level2  residual varriance,ρclass=σu2σu2+σe2=σu2σu2+3.29∗for a two level model.

#### 4.1.1. Age, Residence, Ethnicity, and Religion as Controlled Variables

In the upcoming sections, individual predictors were incorporated to the model to improve the prediction value and develop a best fit model. The first variable input into the model is age of a mother (Table 5). This analysis produces a national level FP utilization prediction that is significantly affected by age expressed in log odds:

(4) $$\begin{array}{c} \log\left(\;\frac{p}{1-p}\;\right)=-1.88+0.0698\left(\;\text{age}-33\;\right); \end{array}$$

which gives a 33-year-old mother to have an average of 24.9 percent FP use:

(5) Piexp⁡β0±2∗σu21+exp⁡β0±2∗σu2=exp⁡−1.1±2∗2.121+exp⁡−1.1±2∗2.12=0.178–0.866.

The coefficient of age implies that FP consumption increases as age increases within a cluster. However, age does not vary significantly between groups, no reduction in group level variance from the null model, with a shift of ICC from 39.8% to 40.2% and between variance from 2.18 to 2.21. A look at the odds ratio values shows that the effect of age is not very strong in affecting FP utilization (Table 5). This might be due to the nature of FP utilization which seems to have two distinct distributions that increases as age increases to the early 30s and then decreases, as can easily be noticed from Table 2.

The addition of religion and ethnicity as predictors further improves our model prediction and reduces the deviance from the null model. In this model Orthodox from religion and Amhara from ethnic category are set to be reference populations. Accordingly, compared to Orthodox religion which has relatively an improved utilization rate in FP, all that other religions have relatively lower utilization rate in FP service. Being Muslim is found to be significantly lower in FP utilization (OR = 0.34, *p* < 0.01). This is supported by many of the available literatures, which put religion as a significant predictor to affect FP use [33–37]. In 1994 in Uganda, according to Korra, due to some religious and community leaders opposition, FP mobilization was reported to be difficult [38].

**Figure 5:**
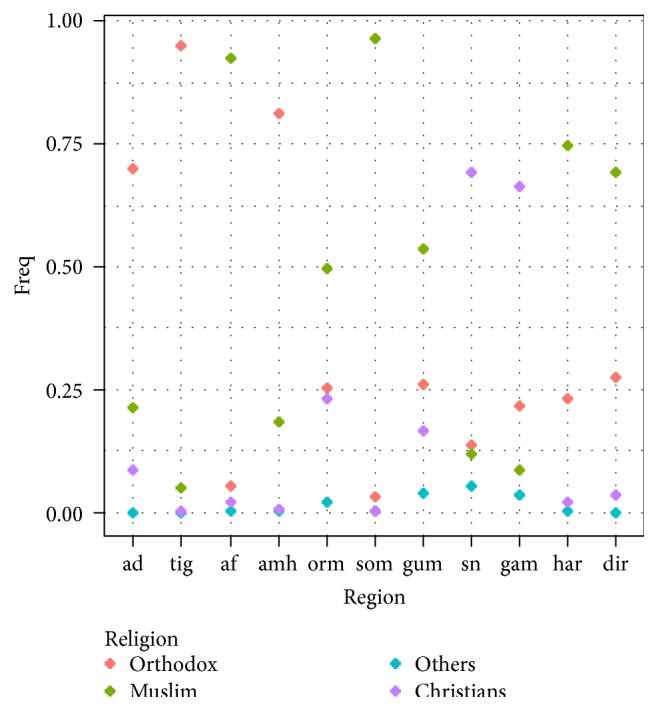
Regional variations of different religions in Ethiopia.

However, by observing Figure 5, one can expect a strong relation between ethnicity and religion. The distribution of Orthodox is much higher in the north, Protestant much higher in Oromiya, Gambela, and SNNP and Oromiya, and Muslim is much higher in afar, Somali, Dire Dawa, Harari, and Oromiya. Taking this relation into consideration, an interaction term is incorporated into the model which shows that the effect of religion almost disappears while the effects of ethnicity still exist, at a cluster level variance of *u*_0*j*_^2^ = 0.72, which explains about 18% (*p* < 0.01).

Hence, religion as one of the structural factors is not found to be statistically significant factor at 5 percent level of significant for FP use; this is in line with the finding from [39], where major religious groups were not found to be significant predictors to affect FP unmet need. However in this study, the effect of religion on FP is highly affected by ethnic differences. Muslims in Oromiya and other nonmajor regions were found to be lower than the other groups to use FP which might be due to differences in culture or service accessablity.

On the other hand, analysis of ethnic differences shows that compared to Amhara, most of the other ethnic categories used in this model have a lower FP practice at a cluster level, even if it is only the other ethnic groups that include Afar, Somali, and Benishangul were found to be significantly lower in FP utilization (OR = 0.31, *p* < 0.01). This finding is in line with a study by Teklu et al. which says that Amhara has the highest decline in fertility by about 24 percent whereas no change in fertility level is observed in Benishangul-Gumuz, Harari, and Gambela regions [25] between the periods 2005 and 2011.

In model four, it was found that urbanities have a much more probability of using FP with predicted values of 35.7% for the rural and 72.3% percent for the urban community, keeping ethnicity and religion constant. Assuming all other predictors constant, the odds of using FP service in rural communities is significantly lower than the urban residents (OR = 0.21, *p* < 0.01). Further evidence supporting this finding may lie in the findings of [34] who found a better access to FP in urban setups. It is understandable to expect that people residing in the urban centers have a better access to information, education, better income, and access to facilities unlike the rural community. This relation was further explored by a study in the rural part of Dauro community, Ethiopia [40], which showed that having better knowledge about modern contraceptive methods, gender equitable attitude, better involvement in decisions related to children, and sociocultural and family relations were statistically significant factors for decision making power of women on the use of modern contraceptive methods in the urban setting.

An intermediate model that includes the combination of age, ethnicity, religion, residence, and an interaction term between ethnicity and religion explains a major part of both the within cluster and between cluster differences. In particular, ethnicity, religion, and rural urban differences explain the major between cluster variations, evidenced by a fall in ICC from 39.8% to 20.2%. The model deviance also improves from 9198 to 8670. This implies that the group effect of these predictors is very strong in justifying the difference in FP use. This might probably be further due to differences in access to infrastructure, human resource, and various cultural differences which is beyond the scope of this study to further disentangle. However, analysis of the structural determinants without including the group level prediction would lead to wrong conclusions as far as big share of the variations is explained by these variables. As opposed to most literatures which say that religion is a determining factor for FP, our finding indicates that the effect of religion on FP is dependent on ethnic variations. A fitted regression line with random intercept model for the intermediate model is shown below:

(6) log⁡p1−p=0.96−0.03age−33−0.3christian−0.92muslim−1.3other.relg−0.21oromo−0.44SNNP−1.04other.eth−1.55rural.

#### 4.1.2. Income and Education

The upcoming section will address the effects of education and income differences on FP services. Among many of the socioeconomic predictors of FP utilization, income is believed to be a key in leading to a better education, access to health facility, access to information, and better living standards. According to EDHS, the overall Gini Coefficient for Ethiopia is 0.23 with least equitable distributions being high in urban setups than rural, in Afar and Gambela than other regions. Keeping ethnicity, religion, and age constant, as can be seen in the following model, education and income were significant determinants for FP. For education, women with secondary and above level were taken as reference group and the FP use of people having no education and primary was compared against the reference. The finding shows that women with no education or primary education were found to have lower odds of FP use (OR = 0.45 (0.11) and OR = 0.72 (0.1), resp.). Similar other studies have showed that the effects of education on FP were higher for women with secondary and above education than with no education [41, 42]. Further evidence supporting the effect of education may lie on the works of Ainsworth et al. [43] that showed the negative relation that the last years of female primary schooling has about half the countries in Africa, while secondary schooling is associated with substantially lower fertility. Both education and income explain a significant portion of the between variation (ICC of 20.2 to 18.9 and to 16.2 by the addition of education and income explanatory variables, resp.) reducing the between variance from 0.83 to 0.61.

On the other hand, taking the richest income group as a reference group, almost all other income categories have a lower proportion of FP service utilization (OR = 0.24 (0.14) for the poorest and OR = 0.39 (0.14) for the poorer). In other words the odds of FP utilization are four times higher in the richest group than the poorest group at a cluster level. The interaction of these predictors did not significantly affect the output of the model, which is supported by the small deviance and almost similar between variances (0.614) which is not significant. However, addition of income variable to the model reduces the variance from 0.76 to 0.63, showing how income has a strong variation within clusters and takes the biggest share in explaining the cluster level differences. The intercluster correlation shows that addition of income predictor reduces the between cluster variation from 18.9 percent to 16.2 percent. The cluster effect still explains more than 16 percent of the variation in FP utilization between the clusters. On this ground, this research appears to validate the view that education and income are strong structural factor that affects the group behavior of communities much more than individual decisions for FP (Table 6). The available evidence on the other hand does not address the effect of education on group level effects. Further research on this have indeed pointed out that women in the richest household wealth quintile, educated women and employed women and urban women, tend to use modern contraception more than other women [34, 44, 45]. Alemayehu et al. 2010, on the other hand, argue that being unemployed, rural and urban setups other than Addis Ababa are found to be risk factors for increased adolescent fertility rate [46]. On the other hand, [42] expressed that the difference in number of children is more than two children per woman between women in the poor and rich wealth groups in 2005 and 2011. The following intermediate model summarizes the effect of income and education while controlling other socioeconomic predictors on FP use:

(7) log⁡p1−p=−0.691+0.074age−33−0.150oromo−0.320SNNP−0.684other−0.053rural−0.378catholic−0.087protest−0.604muslim−0.639trad+0.151primary−0.053second−1.377poorest−0.828poorer−0.715middle−0.366richer.

#### 4.1.3. Media and Health Extension Workers

Another model was developed to evaluate FP knowledge, media utilization, and visit to the household by a health extension worker on FP practice. Media utilization was measured using categorical variables of radio listening, reading newspaper, and watching television. These categorical media level predictors were combined to give a new composite numerical media predicator, named “media” that has six categories. The maximum value of 6 is given for those who have a value of two each for radio listening, television watching, and news reading, exemplified by people who listen radio at least once a week (2 points) and who read newspaper less than once a week (1 point) and who do not watch television (0 point) will have a total of 2 + 1 + 0 = 3/6. Keeping age, ethnicity, religion, and residence constant in the model, FP knowledge is not found to be significantly different for FP use at a cluster level; however, it reduces the between cluster variance from 0.72 to 0.64, which shows a big variation in FP knowledge between clusters while having a strong similarity within the cluster.

Similarly, the input of media at the individual cluster is found to be a significant predictor of FP use with a reduction in group variance from 0.83 to 0.74, which tells that media consumption has significant between cluster variance. The small effect size might tell us the need to well design and develop a very strong media program or the need to expand the reach and frequency of exposure to result in a much pronounced effect. Or it might also tell us the media utilization is relatively weak and innovative ways of reaching the community should be designed to increase access to media and bring behavioral change at a community level. In a similar fashion, addition of health extension visit into the model model (Table 7) shows that FP service is significantly higher with households having a health extension worker visit, with odds ratio of 1.5 (0.07). This predicator has also reduced the between cluster variance from 0.83 to 0.81, with slight between cluster variation, meaning access to health extension service is almost universal to most parts of the cluster in the study area. The final model which contains both health extension and media access indicates that both remain significant predictors, with a between variance of around 17.1 percent. However, the effect of media on FP slightly reduces when community health extension workers visit is kept constant, indicating the importance of both community and media programs to run together for an effective intervention.

#### 4.1.4. Partners Education and Income Status

In the upcoming model the effects of husband education, occupation, relation to household head, household family size, and respondent's occupation were reviewed. As can be seen from Table 8, addition of respondents occupation, taking agriculture as a reference group results in manual workers, professionals, and sales workers to have a significantly higher proportion of FP utilization with OR = 1.34 (0.12), 1.82 (0.16), and 1.2 (0.1), respectively. Similarly inclusion of husband's occupational status shows that manual, professional, and sales workers are much better utilizers of FP service than agricultural workers, most of whom are farmers with OR of 1.55 (0.12), 1.73 (14), and 1.58 (0.1), respectively. Indeed addition of the husband occupational status results in the disappearance of the effects of respondent's occupation on FP utilization. It might be confounded with the husband's occupation but it might tell us that the husband's occupation is much important determinant of FP than the respondents. Furthermore, inclusion of the husband's occupation decreases the deviance from 8571 to 8261, a very big improvement in the model fitness. However, the between cluster variance has only a slight shift from 0.818 to 0.815 which indicates that between cluster variation is weak.

Regarding the effect of husband education of FP, making those with no education as a reference, having elementary and secondary educated partners increases the odds of FP use by 1.53 (0.07) and 1.53 (0.11), respectively. The reduction in the between cluster variance following this predictor indicates the hidden contextual effect that partner's education has on women FP use, beyond its significant effect within the cluster. A similar study in Ethiopia has revealed that partners educational status is an important factor for a woman's FP utilization with OR = 1.32 and 1.5 for a woman with primary and secondary educated partners compared to those who have uneducated partners [34]. They also found that married women who had discussion about contraception with their partners were 2.2 times more likely to use FP compared to those who did not while partner's support to use FP increases the OR to 2.59.

#### 4.1.5. Relation to Household Head and Household Size

Relation of the respondent to the household head is also believed to affect the decision making power of the mother (Table 8). With a similar reasoning, the differences in the distribution of FP practice are found to be significant according to the relation to the household head. Being a wife is strongly linked with FP utilization at an odds ratio of 1.52 (0.1). This indicates that married respondent is sexually active and understands the risk of pregnancy much more than those of sisters and daughters (relatives), with odds ratio of 0.77 (0.33), who might live with their brother or father after divorce or separation from their husband. The same is true for a lady to be a head of the household, as in this reference, who might be widowed or separated and might not have a frequent sexual activity that risks her to pregnancy.

Household size is also well studied to be one of the determining factors for fertility. Our finding demonstrates that households that have a family size of greater than 6 have almost an odds value of 2.3 times FP utilization than those having two and less (OR = 2.3 (0.0)). The same applies for families having 3–5 to use FP much higher than those having 2 and less. Unlike the simple frequency table outcomes, the findings from the model indicate the theoretical assumption that families with higher family size use FP, much more than others; however, the group variance increases. This shows that FP utilization with respect to number of household sizes has a huge variation between clusters. These findings match a study in Pakistan where the number of living children and women educational status [41] were found to be the most prominent predictors of contraception use.

### 4.2. Results and Discussion from Random Slope Model

So far, the multilevel models we have considered have allowed the response probability to vary from group to group by including a group-level random term “*u*_*j*_” in the linear predictor of the model. However, this random term affects only the intercept of the model so that the intercept for group_*j* is *u*_*j*_ + *β*_0_; the effect of each explanatory variable “*x*” is assumed to be the same in each group. We will now consider random slope models that allow the effect of one or more predictors to vary across groups. Indeed practically, predictor variables do not remain constant across clusters as seen in our interclass correlation coefficients. Hence, it is important to fit some variables that are assumed to be varying across different clusters. To start with, it is important to differentiate which variables do vary across clusters and consecutively these variables have to be modeled using the random intercept and random slope model combined. The effect of these predictors in combination with the fixed effects model results will let us know the contribution of cluster level variations for FP service (Refer (2)).

Table 9 summarizes the output from this model.

Variables that were included in the random slope model were generated using cluster level aggregation. Income, education, health extension workers visit, and media access were assumed to vary among clusters. As most of these predictors are categorical variables, a group level predictor is computed taking the proportion of a reference group within that cluster. For education, proportion of members that complete primary education is taken as reference, represented by “prop_primary.” Similarly for income category, the existing five income categories are reclassified as low, middle, and high. In a similar fashion with education, the proportion of high income is taken and its variability among the clusters is taken as a new variable. The proportion of health extension workers visited members and the proportion of people having media access are other two variables included in the model. A total of four variables are included to vary in the level two cluster analysis. Media variable was reclassified into weak and strong media follower based on the existing predictor variable and the proportion of strong media followers is the predictor variable that is included in the level two analyses. The result of the analysis is presented below:

(8) log⁡p1−p=0.80−0.02age−33−0.24oromo−0.36SNNP−0.93other−0.47rural−0.08christ−0.77muslim−1.1other−0.17primary−0.47no.edu−1.62poorest−1.17poorer−0.0.97middle−0.57richer+u1prop.ext+u1prop.primary+u1prop.inc+u1prop.media+uj+eij.

The main intention in this model is to analyze the presence of variation in FP consumption that can be explained by group level predictors. As can be seen from the result, FP utilization significantly varies among groups and the within group variation is better explained by the variation in access to health extension workers visit, media utilization, education, and income variances. In the full model we can notice that, controlling the effect of health extension workers, increasing media coverage has relatively a lower effect on FP utilization. It is an indicator that media utilization has to be supported by community based interventions to become more effective. But it is also very important to note that, though within a cluster the effect of media disappears, it is a very important explanatory variable at a group level, which sounds true in that media access is affected by structural effects than individual level differences where most of the services are limited geographically. Moreover, the design of media approaches might need to be further developed to create impact at a community level. As already explained in the random slope model, keeping constant the richest income group, the odds of FP use are much lower for other categories, a value of 0.28, 0.46, 0.51, and 0.71 sequentially from poorest to richer all being significant at 99% confidence interval. The effect of education in affecting individual level FP service disappears in the final model but its explanation power is very strong at the group level, indicating the importance of improving education coverage for community level effects on FP use.

Observation of the random slope outcome in Table 9 shows that the intercept and slope variances are very high for proportion of health extension workers and proportion of primary school completion. This is found to be statistically significant with a chi square value of 11.26 (2 df) at a *p* value of 0.0036 for proportion of health extension at chi square value of 22.3 (2 df) and p value of 0.00. This evidence explains that the FP consumption variation between clusters is significantly affected by the proportion of health extension workers and variation in educational level of the cluster. Most of the group level predictors show that the level of variation which was high at lower levels of income, education, and media follow-up and visit from health extension workers will have a narrow gap in the level of variation between clusters. This is represented by the negative correlation between the intercept and the group level predicators.

## 5. Conclusion

FP service in Ethiopia has displayed a fast improvement in coverage from 2000 to 2011. However, still fertility is about 5.8 in the rural setup and the level of regional variations in fertility and FP is very height resulting in a net effect that maintains the annual population growth of the population over 2.6, which will lead to a projected 188 million population by 2050, an increase of about 100 million in less than 40 years' time. Though there is limited access to empirical raw data, the availability of DHS data has created access for academicians to investigate the existing factors that affect FP utilization. There is overwhelming evidence corroborating the individual effects of most socioeconomic factors that affect FP use. However, human being has a complex nature of social interaction that affects its individual and communal behaviors. This factor needs to be considered and structural factors that affect individual behavior and group behavior should be targeted differently for a better access to FP. In this paper, the authors put forward the claim that the effects of structural determinants of family planning have both direct and indirect effects at a community level to affect service utilization. In the authors view, inability to understand their indirect contextual effects leads to poor planning and interventions that do not address the primary causes.

This study analyzed 8906 individual women observation from 2011 DHS data using a multilevel modeling that helps understand group level difference. The results show that there is a big variation in FP service utilization both at individual observations and within group (around 39% of the variation in FP use is due to cluster level differences). The fixed effects model in this study propose that age, ethnicity, and residence were found to be important predictor variables for FP use within clusters. The effect of age (OR = 0.97, *p* < 0.01) is a very weak one with small between cluster variation. However, being a rural residence (OR = 0.23 (0.11)) and being in other ethnic groups (Somali, Afar, Benishangul Gumuz, and others) with OR = 0.58 (0.83) were found to be significant predictors for FP use. Unlike the available evidence, religion, on the other hand, seems to have interaction with ethnic group where its importance in explaining the FP utilization gap in Ethiopia disappears (OR = 0.4 (1.21)) after controlling for interaction. However, the cluster level variance shows that all religion, ethnic, and residential factors explain a significant part of the variation in FP use between clusters. The interpretation would imply that these factors have a strong contextual effect on FP too.

In a similar fashion income is found to be a very strong determining factor for FP use (OR = 0.24, ser = 0.129 for the poorest class) and a huge group level effect. Keeping the other factors constant improving income level from the poorest community to a one level poorer scale would improve the odds of FP use from 0.218 to 0.422, an increase of more than 93%. However, education which is believed to improve FP use does not seem to be a strong factor to affect FP use differences within cluster while remaining to be a strong factor for the between cluster differences. These predictors that have strong communal influence need a strong national intervention and individual efforts will be less effective to bring change at a community level.

There are overwhelming evidences that support the role of partners support and his sociodemographic characters that determine FP use. This current research appears to support these ideas, and husband education and occupation were found to be very significant predictors (OR = 2.28 (0.16) for husband education and OR = 1.635 (0.15) for husband occupation). More importantly, husband occupation was found to have a strong between cluster variations that need a policy level intervention to address the gap. Despite these, respondent occupation is found to be insignificant in determining FP utilization, probably because of the low number of employed female workers nationally.

Controlling for the effects of sociodemographic characters, health extension worker visit and media were found to be very significant factors within cluster variations. However, their group variation is very small. The forgoing discussions imply that the FP utilization in Ethiopia is affected by a complex group and individual factors. While ethnicity, rural urban residence, access to media, income level, partners education and occupation, and visit by health extension workers are strong individual level factors that affect FP, still a significant portion of the variation at a group level is explained by income, FP knowledge gap, ethnicity, religion, and husband occupation. Further analysis of these differences at group level supports that the between cluster variation is strongly affected by income, education, and access to media. This research work is conducted to better understand the structural determinants of FP utilization in Ethiopia to design a national based media intervention project that promotes behavioral change for a better FP service utilization at community level. On these grounds the authors would like to recommend the following.

1. Family planning interventions that address contextual differences across communities should be given attention with major emphasis to creating access to ethnic minorities.
2. Improving livelihood and coverage of secondary and above education should be given much attention to bring about a sustainable FP utilization.
3. Media interventions that try to improve FP utilization should be supported with strong community interventions.
4. Improvement in urbanization and diversification of employment options should be a focus for policy planners and implementers to make use of FP programs.
5. Education will improve family planning service utilization when the level of intervention is strong enough to bring community level differences.
6. Empowerment of women needs to be well designed and measures to ensure its implementation should be strengthened to create access to FP service.

## Figures and Tables

**Table 1:** Predictor variables for current family planning utilization.

Sn	Variable	Var. names	Variable labels
	*Dependent variables*		
	Current FP utilization	FP.current	0 = no, 1 = yes
	*Independent variables *[*individual level*]		
	Age	Age	Numeric
	Respondent education	Highest.educ	None, primary, secondary, and higher
	Religion	Religion	Orthodox, Catholic, Protestant, Muslim, traditional, and other
	Respondent occupation	Resp.ocupn	Agricultural, manual, not working, professional, sales, and services
	Income	income	Poorest, poorer, middle, richer, and richest
	Residence type	Residence.type	Urban, rural
	Household size	hhld.number	<2, 3–5, 6–9, ≥10
	Husband occupation	Husband.ocupn	Agricultural, manual, not working, professional, sales, and services
	Husband education	Husband.educ	None, primary, secondary, and higher
	Hhld. relation	Hhld.relation	Head, wife, daughter, sister, other relatives, and others
	Hhld. head sex	Hhld.head.sex	Male, female
	Media exposure	Media exposure	Cont. 0–6
	FP knowledge	FP.knowledge	No, modern, traditional, and folkloric
	*Independent variables* [*cluster level*]		
	Highest income proportion	prop.inc	Proportion of respondents in the richest income group
	Proportion. Media user	prop.media	Proportion of media utilizer above or equal to 3 (respondents)
	Proportion extension visit	prop.xt	Proportion respondents having health extension visit per cluster
	Primary complete proportion	prop.primary	Proportion of respondents who completed primary schools per cluster

**Table 2:** Cross tabulation of current family planning users to sociodemographic characters.

	Unweighted			Weighted			OR	lCI	uCI	*p* value
	Total	FP (yes)	FP (%)	Total	FP (yes)	FP (%)				
*Resp.Education*										
No	5574	1056	18.95%	6061.51	1402.39	23.14%	1.00	NA	NA	NA
Elementary	2536	947	37.34%	2689.17	1002.34	37.27%	1.97	1.79	2.18	0.0000
Secondary	796	492	61.81%	626.63	404.93	64.62%	6.07	5.10	7.22	0.0000
*Region *										
Tigray	870	212	24.37%	552.80	138.24	25.01%	1.00	NA	NA	NA
Afar	703	59	8.39%	78.50	9.23	11.75%	0.40	0.20	0.82	0.0094
Amhara	1185	391	33.00%	2475.65	859.91	34.73%	1.60	1.29	1.97	0.0000
Oromiya	1301	357	27.44%	3675.66	1001.14	27.24%	1.12	0.91	1.38	0.2803
Somali	512	24	4.69%	180.29	8.94	4.96%	0.16	0.08	0.31	0.0000
Ben.-gumuz	785	198	25.22%	108.10	30.19	27.92%	1.16	0.73	1.84	0.5468
SNNP	1207	310	25.68%	1893.47	518.83	27.40%	1.13	0.91	1.41	0.2754
Gambela	717	203	28.31%	44.08	20.32	46.09%	2.56	1.38	4.78	0.0068
Harari	527	191	36.24%	23.32	8.49	36.39%	1.72	0.72	4.08	0.3273
Addis Ababa	575	370	64.35%	313.41	202.02	64.46%	5.44	4.03	7.35	0.0000
Dire Dawa	524	180	34.35%	32.02	12.38	38.66%	1.89	0.90	3.95	0.1430
*Residence *										
Urban	2118	1112	52.50%	1671.49	905.64	54.18%	1.00	NA	NA	NA
Rural	6788	1383	20.37%	7705.81	1904.02	24.71%	0.28	0.25	0.31	0.0000
*Age *										
15–19	727	194	26.69%	721.42	189.67	26.29%	1.00	NA	NA	NA
20–24	1660	556	33.49%	1715.24	617.17	35.98%	1.58	1.30	1.91	0.0000
25–29	2299	675	29.36%	2392.94	733.87	30.67%	1.24	1.03	1.50	0.0257
30–34	1509	442	29.29%	1584.44	532.94	33.64%	1.42	1.17	1.73	0.0005
35–39	1350	387	28.67%	1417.78	425.69	30.03%	1.20	0.98	1.47	0.0772
40–44	808	169	20.92%	868.51	204.59	23.56%	0.86	0.69	1.09	0.2211
45–49	553	72	13.02%	676.97	105.73	15.62%	0.52	0.40	0.68	0.0000
*Religion*										
Orthodox	3360	1342	39.94%	4059.60	1490.70	36.72%	1.00	NA	NA	NA
Muslim	3551	640	18.02%	2863.70	588.94	20.57%	0.45	0.40	0.50	0.0000
Christians	1845	502	27.21%	2284.04	710.99	31.13%	0.78	0.70	0.87	0.0000
Others	150	11	7.33%	169.97	19.03	11.19%	0.22	0.13	0.35	0.0000
*Ethnicity*										
Amhara	2084	934	44.82%	2894.14	1134.43	39.20%	1.00	NA	NA	NA
Oromo	2128	646	30.36%	3229.96	879.24	27.22%	0.58	0.52	0.65	0.0000
SNNP	770	239	31.04%	959.41	231.64	24.14%	0.49	0.42	0.58	0.0000
Other	3874	663	17.11%	2254.18	556.28	24.68%	0.51	0.45	0.57	0.0000
*income*										
Poorest	2272	219	9.64%	1857.11	276.71	14.90%	1.00	NA	NA	NA
Poorer	1476	282	19.11%	1916.25	433.22	22.61%	1.67	1.41	1.97	0.0000
Middle	1407	331	23.53%	1920.16	494.54	25.76%	1.98	1.68	2.33	0.0000
Richer	1436	442	30.78%	1782.34	591.85	33.21%	2.84	2.42	3.34	0.0000
Richest	2315	1221	52.74%	1901.45	1013.34	53.29%	6.52	5.57	7.62	0.0000

**Table 3:** Current family planning utilization with family size and husband characteristics.

*Husband education *										
Secondary	1303	636	48.81%	985.63	531.55	53.93%	3.98	3.45	4.60	0.0000
Elementary	3165	1019	32.20%	3728.81	1193.42	32.01%	1.60	1.45	1.77	0.0000
No	4142	714	17.24%	4451.65	1010.93	22.71%	1.00	NA	NA	NA
*Respondent occupation*										
Agricultural	1881	450	23.92%	2679.10	686.08	25.61%	1	NA	NA	NA
Manual	741	246	33.20%	760.79	290.16	38.14%	1.79	1.51	2.12	0.0000
Not working	4370	1007	23.04%	3981.35	1050.82	26.39%	1.04	0.93	1.16	0.4770
Professional	299	191	63.88%	245.27	157.06	64.04%	5.17	3.93	6.81	0.0000
Sales and services	1519	571	37.59%	1631.09	606.31	37.17%	1.72	1.51	1.96	0.0000
*Husband occupation*										
Agricultural	5894	1173	19.90%	7022.70	1654.42	23.56%	1	NA	NA	NA
Manual	788	402	51.02%	709.44	356.93	50.31%	3.29	2.81	3.84	0.0000
Not working	141	19	13.48%	67.58	13.94	20.63%	0.84	0.47	1.52	0.6670
Professional	627	287	45.77%	469.02	268.22	57.19%	4.33	3.58	5.25	0.0000
Sales and services	1192	490	41.11%	914.95	449.79	49.16%	3.14	2.73	3.61	0.0000
*Under five *										
0	2268	739	32.58%	2283.74	729.99	31.96%	1.00	NA	NA	NA
1	3233	1167	36.10%	3473.73	1312.14	37.77%	1.29	1.16	1.44	0.0000
2	2617	508	19.41%	2874.47	647.33	22.52%	0.62	0.55	0.70	0.0000
3	669	70	10.46%	676.88	109.57	16.19%	0.41	0.33	0.51	0.0000
4	119	11	9.24%	68.48	10.63	15.53%	0.39	0.2	0.76	0.0037
*hhld number*										
<2	657	222	33.79%	589.91	191.71	32.50%	1.00	NA	NA	NA
3–5	3912	1246	31.85%	4248.12	1391.85	32.76%	1.01	0.84	1.22	0.9255
6–9	3752	896	23.88%	4029.96	1100.48	27.31%	0.78	0.65	0.94	0.0092
≥10	585	131	22.39%	509.31	125.61	24.66%	0.68	0.52	0.89	0.0051

**Table 4:** Null model comparison for simple GLS and multilevel modeling.

Fixed model				Mixed model				
	Estimate	*z* value	Pr (>|*z*|)		Estimate	*z* value	Pr (>|*z*|)	
(Intercept)	−0.94 (0.023)	−39.99	<2*e* − 16^*∗∗∗*^	(Intercept)	−1.23 (0.069)	−17.83	<2*e* − 16^*∗∗∗*^	
				Random effects	Variance	Std.Dev.		
Null dev:	10564	8905 df		Clust.	2.178	1.476		
Res.dev.	10564	8905 df		AIC	BIC	logLik	Deviance	df.resid
AIC:	10566			9202.4	9216.6	−4599.2	9198.4	8904

The significance level (*p*) is defined according to the following: “*∗∗∗*” ≤ 0.001, “*∗∗*” ≤ 0.01, “*∗*” ≤ 0.05, and “.” ≤ 0.1.

**Table 5:** Structural determinants of family planning.

Summary (glmer 31)	glmer a		glmer b		glmer 1		glmer 2		glmer 3		glmer 31	
	OR	Coef. (ser)	OR	Coef. (ser)	OR	Coef. (ser)	OR	Coef. (ser)	OR	Coef. (ser^1^)	OR	Coef. (ser)
(Intercept)	0.29	−1.23 (0.07)^*∗∗*^	0.33	−1.1 (0.07)^*∗∗*^	0.58	−0.55 (0.08)^*∗∗*^	0.97	−0.03 (0.09)	2.62	0.96 (0.1)^*∗∗*^	2.11	0.75 (0.1)^*∗∗*^
*I* (age − 33)			0.97	−0.03 (0)^*∗∗*^	0.97	−0.03 (0)^*∗∗*^	0.97	−0.03 (0)^*∗∗*^	0.97	−0.03 (0)^*∗∗*^	0.97	−0.03 (0)^*∗∗*^
Orthodox	Ref.											
Muslim					0.34	−1.09 (0.09)^*∗∗*^	0.37	−1 (0.09)^*∗∗*^	0.40	−0.92 (0.09)^*∗∗*^	0.99	−0.01 (0.15)
relg.Others					0.18	−1.71 (0.35)^*∗∗*^	0.20	−1.61 (0.35)^*∗∗*^	0.25	−1.39 (0.35)^*∗∗*^	0.40	−0.92 (1.21)
Christians					0.62	−0.47 (0.11)^*∗∗*^	0.75	−0.29 (0.11)^*∗∗*^	0.88	−0.13 (0.1)	1.35	0.3 (0.29)
Amhara	Ref.											
Oromo							0.80	−0.22 (0.11)^*∗*^	0.81	−0.21 (0.1)^*∗*^	1.27	0.24 (0.14)
SNNP							0.70	−0.35 (0.15)^*∗*^	0.64	−0.44 (0.14)^*∗∗*^	0.85	−0.17 (0.2)
ethn.Other							0.31	−1.17 (0.11)^*∗∗*^	0.35	−1.04 (0.1)^*∗∗*^	0.52	−0.65 (0.12)^*∗∗*^
ethn.miss							0.49	−0.7 (0.39)	0.53	−0.64 (0.39)	0.58	−0.55 (0.83)
Urban	Ref.											
Rural									0.21	−1.55 (0.11)^*∗∗*^	0.23	−1.47 (0.1)^*∗∗*^
Amhara: Orthodox	Ref.											
Muslim: Oromo											0.27	−1.3 (0.21)^*∗∗*^
relg.other: Oromo											0.48	−0.73 (1.39)
Christian: Oromo											0.47	−0.76 (0.34)^*∗*^
Muslim: SNNP											0.50	−0.69 (0.3)^*∗*^
relg.other: SNNP											0.42	−0.87 (1.39)
Christian: SNNP											0.45	−0.81 (0.38)^*∗*^
Muslim: ethn.other											0.21	−1.58 (0.22)^*∗∗*^
relg.other: ethn.other											0.73	−0.32 (1.34)
Christian: ethn.other											0.64	−0.45 (0.32)
Muslim: ethn.miss											0.61	−0.49 (0.99)
relg.other: ethn.miss											0.00	−9.7 (366.71)
Christian: ethn.miss											0.54	−0.61 (1.1)

	glmer a		glmer b		glmer 1		glmer 2		glmer 3		glmer 31	

AIC	9202		9152		9011		8885		8692		8643	
BIC	9217		9174		9053		8956		8770		8807	
logLik	−4599		−4573		−4499		−4432		−4335		−4299	
Deviance	9198		9146		8999		8865		8670		8597	
var	2.178		2.212		1.743		1.315		0.835		0.706	
ICC	0.398		0.402		0.346		0.286		0.202		0.177	

The significance level (*p*) is defined according to the following: “*∗∗∗*” ≤ 0.001, “*∗∗*” ≤ 0.01, “*∗*” ≤ 0.05, and “.” ≤ 0.1.

^1^Ser: the values in the bracket following the odds ratio values that represent standard error!

**Table 6:** Income and education on FP service utilization controlling for other predictors.

>summary (glmer 52) $coef.	glmer 3		glmer 4		glmer 5		glmer 51	
	OR	Coef. (ser)	OR	Coef. (ser)	OR	Coef. (ser)	OR	Coef. (ser)
(Intercept)	2.62	0.96 (0.1)^*∗∗*^	3.43	1.23 (0.12)^*∗∗*^	2.74	1.01 (0.1)^*∗∗*^	3.36	1.21 (0.11)^*∗∗*^
*I* (age − 33)	0.97	−0.03 (0)^*∗∗*^	0.98	−0.02 (0)^*∗∗*^	0.98	−0.03 (0)^*∗∗*^	0.98	−0.02 (0)^*∗∗*^
Orthodox								
Muslim	0.40	−0.92 (0.09)^*∗∗*^	0.43	−0.83 (0.09)^*∗∗*^	0.41	−0.9 (0.09)^*∗∗*^	0.43	−0.83 (0.09)^*∗∗*^
relg.other	0.25	−1.39 (0.35)^*∗∗*^	0.26	−1.35 (0.35)^*∗∗*^	0.31	−1.17 (0.35)^*∗∗*^	0.31	−1.16 (0.35)^*∗∗*^
Christian	0.88	−0.13 (0.1)	0.86	−0.16 (0.1)	0.94	−0.06 (0.1)	0.92	−0.09 (0.1)
Amhara								
Oromo	0.81	−0.21 (0.1)^*∗*^	0.83	−0.19 (0.1)	0.77	−0.26 (0.1)^*∗*^	0.78	−0.24 (0.1)^*∗*^
SNNP	0.64	−0.44 (0.14)^*∗∗*^	0.67	−0.4 (0.14)^*∗∗*^	0.61	−0.49 (0.14)^*∗∗*^	0.63	−0.46 (0.14)^*∗∗*^
Ethn.other	0.35	−1.04 (0.1)^*∗∗*^	0.36	−1.03 (0.1)^*∗∗*^	0.38	−0.96 (0.1)^*∗∗*^	0.39	−0.95 (0.1)^*∗∗*^
Ethn.miss	0.53	−0.64 (0.39)	0.58	−0.54 (0.39)	0.54	−0.61 (0.39)	0.59	−0.53 (0.39)
Urban								
Rural	0.21	−1.55 (0.11)^*∗∗*^	0.29	−1.23 (0.11)^*∗∗*^	0.53	−0.64 (0.14)^*∗∗*^	0.60	−0.51 (0.14)^*∗∗*^
Secondary								
Educnno			0.45	−0.8 (0.11)^*∗∗*^			0.53	−0.63 (0.11)^*∗∗*^
Elementary			0.72	−0.33 (0.1)^*∗∗*^			0.77	−0.26 (0.1)^*∗*^
Richest								
Poorest					0.21	−1.58 (0.14)^*∗∗*^	0.24	−1.41 (0.14)^*∗∗*^
Poorer					0.34	−1.09 (0.14)^*∗∗*^	0.39	−0.95 (0.14)^*∗∗*^
Inc.middle					0.41	−0.89 (0.13)^*∗∗*^	0.47	−0.76 (0.13)^*∗∗*^
Incomericher					0.59	−0.53 (0.12)^*∗∗*^	0.65	−0.43 (0.12)^*∗∗*^

		glmer 3	glmer 4		glmer 5		glmer 51	

AIC		8692	8626		8556		8517	
BIC		8770	8718		8663		8638	
logLik		−4335	−4300		−4263		−4242	
Deviance		8670	8600		8526		8483	
Var		0.835	0.764		0.635		0.610	
ICC		.202	0.189		0.162		0.156	

The significance level (*p*) is defined according to the following: “*∗∗∗*” ≤ 0.001, “*∗∗*” ≤ 0.01, “*∗*” ≤ 0.05, and “.” ≤ 0.1.

**Table 7:** Structural determinants of family planning in Ethiopia, effect of access to media, and home visit by HEW.

(glmer 73) $coef.	glmer 3		glmer 6		glmer 71		glmer 73	
	OR	Coef. (ser)	OR	Coef. (ser)	OR	Coef. (ser)	OR	Coef. (ser)
(Intercept)	2.62	0.96 (0.1)^*∗∗*^	1.43	0.36 (0.12)^*∗∗*^	2.43	0.89 (0.1)^*∗∗*^	0.00	−15.83 (508.68)
*I* (age − 33)	0.97	−0.03 (0)^*∗∗*^	0.98	−0.02 (0)^*∗∗*^	0.97	−0.03 (0)^*∗∗*^	0.98	−0.02 (0)^*∗∗*^
Orthodox								
Muslim	0.40	−0.92 (0.09)^*∗∗*^	0.43	−0.85 (0.09)^*∗∗*^	0.40	−0.91 (0.09)^*∗∗*^	0.94	−0.8 (0.08)^*∗*^
relg.other	0.25	−1.39 (0.35)^*∗∗*^	0.28	−1.28 (0.35)^*∗∗*^	0.25	−1.38 (0.35)^*∗∗*^	1.08	−1.13 (0.36)^*∗∗*^
christians	0.88	−0.13 (0.1)	0.92	−0.09 (0.1)	0.91	−0.1 (0.1)	0.94	−0.06 (0.1)^*∗∗*^
Amhara	Ref							
Oromo	0.81	−0.21 (0.1)^*∗*^	0.80	−0.22 (0.1)^*∗*^	0.81	−0.21 (0.1)^*∗*^	0.79	−0.23 (0.1)^*∗*^
SNNP	0.64	−0.44 (0.14)^*∗∗*^	0.64	−0.45 (0.14)^*∗∗*^	0.65	−0.44 (0.14)^*∗∗*^	0.64	−0.45 (0.14)^*∗∗*^
ethn.other	0.35	−1.04 (0.1)^*∗∗*^	0.36	−1.02 (0.1)^*∗∗*^	0.35	−1.05 (0.1)^*∗∗*^	0.38	−0.97 (0.1)^*∗∗*^
Urban	Ref							
Rural	0.21	−1.55 (0.11)^*∗∗*^	0.31	−1.16 (0.11)^*∗∗*^	0.21	−1.56 (0.11)^*∗∗*^	0.45	−0.81 (0.09)^*∗∗*^
Media			1.21	0.19 (0.02)^*∗∗*^			1.19	.17 (0.022)^*∗∗*^
xt.visit					1.50	0.41 (0.07)^*∗∗*^	1.39	0.33 (0.07)^*∗∗*^

			glmer 6		glmer 71		glmer 73	

AIC			8561		8661		8463	
BIC			8639		8746		8562	
logLik			−4270		−4319		−4217	
Deviance			8539		8637		8435	
var			0.749		0.815		0.680	
ICC			0.185		0.199		0.171	

The significance level (*p*) is defined according to the following: “*∗∗∗*” ≤ 0.001, “*∗∗*” ≤ 0.01, “*∗*” ≤ 0.05, and “.” ≤ 0.1.

**Table 8:** Structural determinants of FP and partner's related predictors.

>summary (glmer 84)	glmer 3		glmer 80		glmer 81		glmer 82		glmer 83	
	OR	Coef. (ser)	OR	Coef. (ser)	OR	Coef. (ser)	OR	Coef. (ser)	OR	Coef. (ser)
(Intercept)	2.62	0.96 (0.1)^*∗∗*^	2.28	0.83 (0.13)^*∗∗*^	1.67	0.51 (0.15)^*∗∗*^	1.27	0.24 (0.16)	0.90	−0.11 (0.19)
*I* (age − 33)	0.97	−0.03 (0)^*∗∗*^	0.97	−0.03 (0)^*∗∗*^	0.98	−0.03 (0)^*∗∗*^	0.98	−0.02 (0)^*∗∗*^	0.98	−0.03 (0)^*∗∗*^
Orthodox			ref							
Muslim	0.40	−0.92 (0.09)^*∗∗*^	0.42	−0.86 (0.09)^*∗∗*^	0.42	−0.87 (0.09)^*∗∗*^	0.44	−0.82 (0.09)^*∗∗*^	0.44	−0.82 (0.09)^*∗∗*^
relg.Others	0.25	−1.39 (0.35)^*∗∗*^	0.26	−1.35 (0.35)^*∗∗*^	0.25	−1.4 (0.37)^*∗∗*^	0.24	−1.44 (0.37)^*∗∗*^	0.24	−1.41 (0.37)^*∗∗*^
Christians	0.88	−0.13 (0.1)	0.91	−0.09 (0.1)	0.91	−0.09 (0.11)	0.87	−0.14 (0.11)	0.86	−0.15 (0.11)
Amhara			ref							
Oromo	0.81	−0.21 (0.1)^*∗*^	0.80	−0.23 (0.1)^*∗*^	0.77	−0.26 (0.11)^*∗*^	0.72	−0.32 (0.11)^*∗∗*^	0.72	−0.34 (0.11)^*∗∗*^
SNNP	0.64	−0.44 (0.14)^*∗∗*^	0.63	−0.47 (0.14)^*∗∗*^	0.58	−0.55 (0.14)^*∗∗*^	0.55	−0.6 (0.15)^*∗∗*^	0.54	−0.61 (0.14)^*∗∗*^
ethn.other	0.35	−1.04 (0.1)^*∗∗*^	0.35	−1.06 (0.1)^*∗∗*^	0.32	−1.13 (0.1)^*∗∗*^	0.32	−1.14 (−0.1)^*∗∗*^	0.32	−1.13 (0.1)^*∗∗*^
Ethn.miss	0.53	−0.64 (0.39)	0.39	−0.95 (0.46)^*∗*^	0.40	−0.91 (0.46)^*∗*^	0.37	−1 (0.47)^*∗*^	0.37	−1 (0.47)^*∗*^
Urban			ref							
Rural	0.21	−1.55 (0.11)^*∗∗*^	0.24	−1.44 (0.11)^*∗∗*^	0.32	−1.13 (0.13)^*∗∗*^	0.35	−1.04 (0.13)^*∗∗*^	0.36	−1.01 (0.13)^*∗∗*^
resp. Agriculture			ref							
resp. manual			1.34	0.29 (0.12)^*∗*^	1.26	0.23 (0.12)	1.25	0.22 (0.13)	1.27	0.24 (0.12)
resp. notworking			0.92	−0.08 (0.08)	0.88	−0.12 (0.09)	0.88	−0.13 (0.09)	0.87	−0.14 (0.09)
resp. proff			1.82	0.6 (0.16)^*∗∗*^	1.46	0.38 (0.18)^*∗*^	1.39	0.33 (0.18)	1.40	0.34 (0.18)
resp. sales and services			1.24	0.21 (0.1)^*∗*^	1.22	0.2 (0.1)	1.20	0.18 (0.1)	1.22	0.2 (0.1)
Husb.Agriculture			ref							
Husb.mannual					1.55	0.44 (0.12)^*∗∗*^	1.45	0.37 (0.13)^*∗∗*^	1.54	0.43 (0.13)^*∗∗*^
Husb.notworking					0.85	−0.16 (0.29)	0.79	−0.23 (0.29)	0.91	−0.09 (0.29)
Husb.proff					1.73	0.55 (0.14)^*∗∗*^	1.54	0.43 (0.15)^*∗∗*^	1.58	0.46 (0.15)^*∗∗*^
husb.sales and services					1.58	0.46 (0.1)^*∗∗*^	1.52	0.42 (0.11)^*∗∗*^	1.58	0.46 (0.11)^*∗∗*^
husb.educnno										
husb.elementary							1.53	0.42 (0.07)^*∗∗*^	1.52	0.42 (0.07)^*∗∗*^
husb.secondary							1.53	0.43 (0.11)^*∗∗*^	1.63	0.49 (0.11)^*∗∗*^
hhld.rltnhead			ref							
hhld.rltnwife									1.52	0.42 (0.1)^*∗∗*^
hhld.rltnrelative									0.77	−0.26 (0.33)
hhld.rltnOthers									0.83	−0.19 (0.15)
hhld.cat <2			ref							
hhld.cat 3–5									1.92	0.65 (0)^*∗∗*^
hhld.cat 6–9									2.34	0.85 (0)^*∗∗*^
hhld.cat ≥10									2.32	0.84 (0)^*∗∗*^

			glmer 80		glmer 81		glmer 82		glmer 83	

AIC			8571		8261		8137		8099	
BIC			8677		8395		8285		8268	
logLik			−4270		−4111		−4048		−4025	
Deviance			8541		8223		8095		8051	
Var			0.818		0.815		0.764		0.749	
ICC			0.199		0.199		0.188		0.185	

The significance level (*p*) is defined according to the following: “*∗∗∗*” ≤ 0.001, “*∗∗*” ≤ 0.01, “*∗*” ≤ 0.05, and “.” ≤ 0.1.

**Table 9:** Random slope outcomes.

Random slope model	glmer 3		glmer 740		glmer 7410		glmer 7420		glmer 7430		glmer 751	
	OR	Coef. (ser)	OR	Coef. (ser)	OR	Coef. (ser)	OR	Coef. (ser)	OR	Coef. (ser)	OR	Coef. (ser)
(Intercept)	2.62	0.96 (0.1)^*∗∗*^	2.48	0.91 (0.1)^*∗∗*^	2.74	1.01 (0.09)^*∗∗*^	1.40	0.34 (0.11)^*∗∗*^	3.44	1.24 (0.12)^*∗∗*^	2.23	0.8 (0.14)^*∗∗*^
*I* (age − 33)	0.97	−0.03 (0)^*∗∗*^	0.97	−0.03 (0)^*∗∗*^	0.97	−0.03 (0)^*∗∗*^	0.98	−0.02 (0)^*∗∗*^	0.98	−0.02 (0)^*∗∗*^	0.98	−0.02 (0)^*∗∗*^
Orthodox												
Muslim	0.4	−0.92 (0.09)^*∗∗*^	0.42	−0.87 (0.09)^*∗∗*^	0.40	−0.91 (0.09)^*∗∗*^	0.44	−0.83 (0.09)^*∗∗*^	0.43	−0.83 (0.09)^*∗∗*^	0.46	−0.77 (0.09)^*∗∗*^
relg.Others	0.25	−1.39 (0.35)^*∗∗*^	0.26	−1.35 (0.35)^*∗∗*^	0.31	−1.18 (0.36)^*∗∗*^	0.29	−1.25 (0.35)^*∗∗*^	0.26	−1.35 (0.35)^*∗∗*^	0.33	−1.11 (0.35)^*∗∗*^
Christians	0.88	−0.13 (0.1)	0.91	−0.1 (0.1)	0.92	−0.08 (0.1)	0.92	−0.08 (0.1)	0.86	−0.15 (0.1)	0.92	−0.08 (0.1)
Amhara	ref											
Oromo	0.81	−0.21 (0.1)^*∗*^	0.78	−0.25 (0.1)^*∗*^	0.80	−0.23 (0.1)^*∗*^	0.85	−0.17 (0.1)	0.82	−0.2 (0.1)	0.79	−0.24 (0.1)^*∗*^
SNNP	0.64	−0.44 (0.14)^*∗∗*^	0.62	−0.48 (0.14)^*∗∗*^	0.65	−0.44 (0.14)^*∗∗*^	0.71	−0.34 (0.14)^*∗*^	0.66	−0.41 (0.14)^*∗∗*^	0.69	−0.36 (0.13)^*∗∗*^
ethn.other	0.35	−1.04 (0.1)^*∗∗*^	0.34	−1.07 (0.1)^*∗∗*^	0.39	−0.94 (0.1)^*∗∗*^	0.39	−0.95 (0.1)^*∗∗*^	0.35	−1.04 (0.1)^*∗∗*^	0.39	−0.93 (0.1)^*∗∗*^
Urban	ref											
Rural	0.21	−1.55 (0.11)^*∗∗*^	0.22	−1.5 (0.1)^*∗∗*^	0.55	−0.59 (0.13)^*∗∗*^	0.30	−1.2 (0.1)^*∗∗*^	0.29	−1.22 (0.11)^*∗∗*^	0.62	−0.47 (0.13)^*∗∗*^
Richest												
Poorest					0.20	−1.62 (0.14)^*∗∗*^					0.28	−1.28 (0.15)^*∗∗*^
Poorer					0.31	−1.17 (0.14)^*∗∗*^					0.42	−0.87 (0.14)^*∗∗*^
Middle					0.38	−0.97 (0.13)^*∗∗*^					0.50	−0.69 (0.14)^*∗∗*^
Richer					0.56	−0.57 (0.12)^*∗∗*^					0.69	−0.38 (0.12)^*∗∗*^
xt.visit			1.52	0.42 (0.07)^*∗∗*^							1.40	0.33 (0.07)^*∗∗*^
Media							1.20	0.19 (0.02)^*∗∗*^			1.08	0.08 (0.02)^*∗∗*^
Secondary												
Educnno									0.45	−0.8 (0.11)^*∗∗*^	0.62	−0.47 (0.12)^*∗∗*^
Elementary									0.72	−0.33 (0.1)^*∗∗*^	0.85	−0.17 (0.11)

			glmer 740		glmer 7410		glmer 7420		glmer 7430		glmer 751	

AIC			8597		8496		8536		8580		8408	
BIC			8689		8609		8628		8679		8635	
logLik			−4285		−4232		−4255		−4276		−4172	
Deviance			8571		8464		8510		8552		8344	
Var			1.463		1.124		1.295		0.937		1.352	
prop.primary			4.574		0.255		0.987		2.783		0.558	
prop.xt											0.684	
prop.media											1.460	
prop.rich											1.739	

The significance level (*p*) is defined according to the following: “*∗∗∗*” ≤ 0.001, “*∗∗*” ≤ 0.01, “*∗*” ≤ 0.05, and “.” ≤ 0.1.
